# Defects at the Posttranscriptional Level Account for the Low TCR*ζ* Chain Expression Detected in Gastric Cancer Independently of Caspase-3 Activity

**DOI:** 10.1155/2020/1039458

**Published:** 2020-11-28

**Authors:** Ana Aguinaga-Barrilero, Patricia Castro-Sánchez, Ignacio Juárez, Alberto Gutiérrez-Calvo, Noelia Rodríguez-Pérez, Adela Lopez, Remedios Gómez, José M. Martin-Villa

**Affiliations:** ^1^Inmunología, Facultad de Medicina, Universidad Complutense de Madrid, Madrid, Spain; ^2^Servicio de Cirugía General y Aparato Digestivo, Hospital Universitario Príncipe de Asturias, Alcalá de Henares, Madrid, Spain; ^3^Instituto de Investigación Sanitaria Gregorio Marañón (IISGM), Madrid, Spain

## Abstract

**Background:**

Reduced TCR*ζ* chain surface has been reported in T cells from patients with different inflammatory conditions and cancer. However, the causes of this diminished expression in cancer remain elusive.

**Methods:**

T cell-enriched populations of blood or tissue (tumoral and nontumoral) origin from 44 patients with gastric adenocarcinoma and 33 healthy subjects were obtained. Samples were subjected to cytofluorimetry, Western blot analysis, TCR*ζ* cDNA sequencing experiments, measurement of TCR*ζ* mRNA levels, and caspase-3 activity assays.

**Results:**

Cytofluorimetry revealed a decreased TCR*ζ* expression in T cells of patients, assessed either as percentage of cells expressing this chain (blood: control subjects 99.8 ± 0.1%, patients 98.8 ± 1.1%*P* < 0.001; tissue: control subjects 96.7 ± 0.9%, patients tumoral tissue 67.9 ± 27.0%, patients nontumoral tissue 82.8 ± 12.6%, *P* = 0.019) or mean fluorescence intensity (MFI) value (blood: control subjects 102.2 ± 26.0; patients 58.0 ± 12.3, *P* = 0.001; tissue: control subjects 99.4 ± 21.4; patients tumoral tissue 41.6 ± 21.4; patients nontumoral tissue 62.3 ± 16.6, *P* = 0.001). Other chains pertaining to the TCR-CD3 complex (CD3*ε*) showed no significant differences (MFI values). Subsequent TCR*ζ* cDNA sequencing experiments or measurements of TCR*ζ* mRNA levels disclosed no differences between patients and control subjects. Evaluation of caspase-3 activity showed higher levels in T cell extracts of patients, and this activity could be decreased by 70% with the use of the inhibitor Ac-DEVD-FMK, although CD3*ζ* expression levels did not recover.

**Conclusions:**

These results further place the defect responsible for the low TCR*ζ* expression in cancer at the posttranscriptional level and suggests contrary to what has been proposed in other pathologies that elevated caspase-3 activity is not the causative agent.

## 1. Introduction

Tumoral antigens expressed upon neoplastic transformation may be targets of cells of the immune system, such as T lymphocytes. In fact, tumor-specific T cells are found in the circulation of patients with cancer [1], or infiltrating the tumor tissue (tumor-infiltrating lymphocytes, TIL), exerting immunosurveillance functions [2]. Recent immunotherapeutic approaches to cancer and tumour dissemination have focused on limiting physiological T cell inactivation (via CD80-CTLA4 or PD1-PDL1 interactions) to enhance T cell activation to tumoral cells [3].

To accomplish this task, T cells require fully functional signaling machinery, able to detect the presence of “*de novo*” antigens and carry out an adequate response. However, several phenotypic or functional T cell alterations have been described in patients with cancer, and, therefore, T cells are unable to keep tumoral cells at bay [4]. Downregulation of T cell receptor- (TCR-) associated cell surface proteins, such as TCR*ζ* (CD247) [5], is one of such alterations. This downregulation was found not only in cancer but also in other inflammatory conditions, as systemic lupus erythematosus, (SLE [6, 7]) amongst others.

The TCR*ζ* chain is a 16 kDa protein expressed by NK, NKT, and T cells and contains a long cytoplasmic tail with three immunoreceptor tyrosine-based activation motifs (ITAM). It is involved in signal transduction and is crucial for the correct function of T lymphocytes [8], and therefore, its downregulation certainly limits the ability of T cells to respond to tumor antigens.

The causes whereby T cells from patients express low TCR*ζ* levels remain unclear, and several mechanisms have been proposed: lysosomal degradation [9], alternative mRNA splicing [10], presence of peroxide metabolites [11], Fas-FasL interactions [12], or increased caspase-3 activity [7]. Our group previously suggested that the defect seemed inherent to T cells, since Herpesvisrus saimiri- (HVS-) transformed T cell lines, grown *in vitro* for several months, maintained this TCR*ζ* defective expression [13–16].

We wished to confirm this defect in freshly isolated T cells of blood and gastric origin from patients with gastric adenocarcinoma. Once the TCR*ζ* expression was assessed, TCR*ζ* cDNA sequencing and measurement of TCR*ζ* mRNA levels were carried out. Furthermore, caspase-3 activity was also quantitated in the absence or presence of the caspase inhibitor Ac-DEVD-FMK, to determine whether the defect could be placed at the genomic, transcriptional, or posttranscriptional level.

## 2. Material and Methods

### 2.1. Patients

Forty-four patients with gastric adenocarcinoma, classified according to the Japanese Research Society for Gastric Cancer criteria [17] were included in this study. Patients (30 men, 14 women, median age: 67 yrs. range 42 yrs.-89 yrs.) were submitted by the Department of General Surgery (Servicio de Cirugía General y Aparato Digestivo) of the Hospital Príncipe de Asturias, Alcalá de Henares. As a control group, blood samples were obtained from nineteen healthy unrelated individuals (10 men, 9 women, mean age: 28 yrs. range 24 yrs-50 yrs) who were included. In addition, blood and tissue samples were obtained from further 14 individuals (5 men; 9 women; median age: 48 yrs. range 30–61) who underwent gastric surgery for reasons other than cancer (morbid obesity).

### 2.2. Preparation of Blood Samples

Blood from patients was drawn on the day of surgery using EDTA-containing tubes, prior to any manipulation of the patient.

Purified T cells were isolated from the blood samples using a T cell enrichment cocktail (RosetteSep, Stem Cell technologies), following manufacturer's indications. In brief, 20 mL whole blood was incubated with 500 *μ*L of the above reagent for 20 minutes at room temperature. Then, the suspension was subjected to centrifugation (45 min, 900 g) on a lymphoprep cushion, and T cells were isolated at the interphase and washed twice with phosphate-buffered saline (PBS). T cell purity achieved was always higher than 95%.

### 2.3. Preparation of Tissue Samples

Tissue samples were obtained upon surgery and transported to the laboratory in RPMI 1640 medium with antibiotic and minced in a Petri dish with HBSS in 0.5 cm fragments and treated with DTT for 30 minutes with gentle agitation. After two washing steps with PBS, samples were treated for further 40 minutes with HBSS 1 mM EDTA. Supernatant, rich in intraepithelial lymphocytes, was then collected and kept on ice until use. Next, to isolate lamina propria lymphocytes, pieces were washed with PBS to eliminate DTT and EDTA and subjected to collagenase digestion (50 U/mL in RPMI) for 90 minutes at 37°C with gentle agitation. Suspensions thus obtained, along with the cells previously kept on ice, were filtered through a 70 *μ*m mesh and washed twice. Then, cells were layered onto a Percoll gradient (20%-44%-67%) and centrifuged. Lymphocytes were collected at the 44%-67% interphase and washed twice with PBS. Finally, a T cell enrichment step was carried out using a Pan T cell isolation kit (MiltenyiBiotec).

T cell enriched populations thus obtained were used for cytometry, mRNA isolation, cDNA sequencing, or quantitative RT-PCR (qRT-PCR) or caspase-3 activity experiments.

Blood and tissue samples from control subjects were treated likewise.

### 2.4. Flow Cytometry

**Figure 1:**
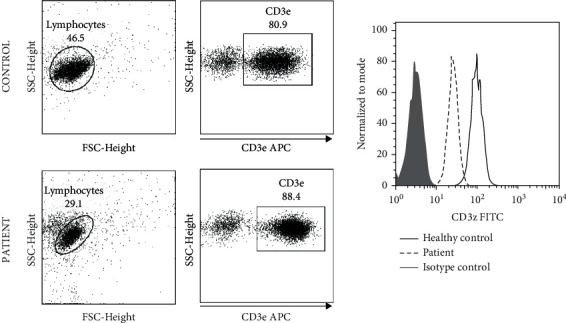
Representative DotPlots and histograms of the CD3z gating and MFI analysis. A two-gate strategy was employed, using forward scatter (FSC) versus side scatter (SSC) to characterize the previously isolated lymphocytes and CD3*ε* to determine the T lymphocytes. CD3*ζ* MFI were determined within the CD3*ε*-positive population.

CD3*ε* (surface) and TCR*ζ* (intracellular) expression was assessed by flow cytometry. The analysis of the expression of TCR*ζ* was done using two different monoclonal antibodies. Purified T cells (2 × 10^5^) were incubated with an APC-conjugated CD3*ε*-specific monoclonal antibody (clone UCHT1, BD) for 30 min at 4°C, washed twice, and treated with Cellfix solution (Becton Dickinson) for 10 min at 4°C. Cells were then permeabilized with 0.5% BSA-0.1% saponin in PBS and incubated either with a primary unconjugated (clone G3, Serotec) or a FITC-conjugated (clone 6B10.2, BioLegend) TCR*ζ*-specific monoclonal antibody for 30 min at room temperature (RT). After two washing steps with permeabilization solution, cells were either resuspended in FACS flow or, when required, incubated with a secondary polyclonal anti-mouse FITC-conjugated antibody (Serotec) for further 30 minutes in the dark. Finally, and after two further washing steps, cells were resuspended in Facs Flow (Becton Dickinson). Isotype-matched monoclonal antibodies were used as negative controls in all cases. A minimum of 50000 cells were analyzed per sample and gated to exclude nonviable cells. Fluorescence intensities above the upper limit of the negative control distribution were considered positive. In Figure 1, representative dot plots and histograms of CD3*ζ* gating and MFI analysis are shown.

### 2.5. Western Blot

Enriched T cells (5 × 10^6^) of blood origin were used. Cells were pelleted and resuspended in 50 *μ*L of lysis buffer supplemented with protease inhibitors, 5 mM NaF, 0.2 mM sodium vanadate, and 1 mM phenylmethylsulfonyl fluoride (PMSF) for two hours on ice. Then, the suspension was centrifuged at 14000 rpm for 5 minutes, and the supernatant collected. 40 *μ*g of protein extract were boiled (100°C, 5 min) and resolved on a 12% polyacrylamide gel in reducing conditions (100 V, 90 min) and transferred (30 V, 60 min) onto a polyvinylidene fluoride (PDVF) membrane. Membrane was then blocked in nonfat milk, followed by incubation with a primary monoclonal antibody (Santa Cruz) TCR*ζ*- or a CD3*ε*-specific (overnight incubation, 4°C). After several washing steps, membranes were incubated with a goat anti-mouse horseradish peroxidase- (HRP-) conjugated secondary antibody (Santa Cruz) and developed with ECL reagents. Bands were quantitated by densitometry and the optical density (OD) of the TCR*ζ* band normalized against the CD3*ε* band (used as a load control protein) for each sample.

### 2.6. RNA and DNA Extraction

RNA and DNA extraction from purified T cells was achieved using TRIzol reagent (Invitrogren). This reagent allows sequential purification of RNA and then DNA from a single sample.

### 2.7. cDNA Sequencing

Total RNA was retrotranscribed to cDNA using the First-Strand cDNA synthesis kit (Roche). TCR*ζ*-specific cDNA amplification was then carried out. To amplify the whole cDNA transcript, five pairs of primers were used (see Table 1). Amplification conditions were as follows: 94°C 5 minutes; 94°C 30 seconds; 55°C 30 seconds; 72°C 30 seconds (35 cycles); final extension step 72°C 10 minutes. PCR products were run in an agarose gel, eluted from the gel (Quiagen Elute Gel extraction kit 250), and sequenced in the DNA sequencing facility of the CSIC, Madrid (Secugen). Analysis of the sequences obtained was done using the Chromalite Software (v2.0.1 Technelysium Pty Ltd) and compared to already deposited sequences at the NCBI database.

### 2.8. TCR*ζ*-mRNA Expression Levels

Total RNA was treated with DNAase (Ambion) to achieve DNA-free RNA sample, and it was then retrotranscribed to cDNA, as before. mRNA levels were measured (at the Genomic Unit, Parque Científico de Moncloa, UCM) by qRT-PCR using a TaqMan Gene Expression assay kit and TCR*ζ*-specific primers (Applied Biosystems). As reference, and to normalize TCR*ζ* levels, 18S rRNA amplification was also carried out.

### 2.9. Measurement of Caspase-3 Activity

Caspase-3 activity was measured in blood T lymphocytes with the Caspase-3 Cellular Assay Kit PLUS (Enzo Life Sciences) following manufacturer's instructions. The assay is based on the cleavage of the substrate DEVD bound to the chromophore *p*-nitroanilide (*p*NA) by caspase-3. Briefly, cells were lysed, and lysates (10 *μ*L) were incubated with the substrate DEVD-*p*Na (200 *μ*M) at 37°C. Caspase-3 activity was measured by reading the absorbance (at 405 nm) of the samples every 15 minutes for 90 min. Lysate protein content was determined by Bradford assay, and caspase-3-specific activity was calculated as pmol of p-Na produced per minute per *μ*g of protein (pmol p-Na/min/*μ*g protein). To assess the specificity of the assay, caspase-3 was inhibited by incubating the cells for 8 hours in a CO_2_ incubator with the inhibitor Z-Asp-Glu-Val-Asp-FMK (DEVD, Calbiochem) 50 *μ*M prior to lysis, and caspase-3 activity was measured as before.

### 2.10. Statistical Analysis

Results obtained are shown as mean value ± standard deviation (s.d.). Mann-Whitney *U*-test was used to carry out comparisons between patient and control groups, with the SPSS (19.0 version) software. In experiments where more than two groups were compared, Kruskal-Wallis test followed by post hoc test for pairwise comparison of subgroups, using the MedCalc software. A *P* value less than 0.05 was considered significant.

## 3. Results

### 3.1. Flow Cytometry

#### 3.1.1. Blood-Derived T Cells

Purified T cells were obtained from 21 patients and 28 control subjects. The percentage of T cells expressing TCR*ζ* rose to 99.8 ± 0.1% in control subjects and 98.8 ± 1.1% in patients (*P* < 0.05). When MFI values are considered TCR*ζ*, MFI is significantly reduced in patients (102.2 ± 26.0 vs. 58.0 ± 12.3; *P* = 0.01). This lower MFI was found on both CD4+ and CD8+ T cells, showing that the defect affects all T cells and is not lineage-specific. As for the CD3*ε* MFI value, we detected just a minor, not significant, reduction in the level of the CD3*ε* chain on the cell surface in gastric cancer compared to healthy subjects.

Thus, TCR*ζ* is reduced in T cells from patients with cancer. This impaired expression does not affect other constituents of the T cell receptor (TCR) complex, such as CD3*ε*.

#### 3.1.2. Mucosa-Derived T Cells

TCR*ζ* and CD3*ε* expression was also analyzed in tissue-derived (tumoral or nontumoral) T lymphocytes from patients with gastric cancer (*n* = 5) and control individuals (*n* = 5) subjected to surgery for reasons other than cancer. The percentage of TCR*ζ*-expressing cells is significantly diminished (Kruskall-Wallis test *P* = 0.019) in tumoral tissue-derived T cells from patients (67.9 ± 27.0%) when compared to nontumoral tissue-derived cells from patients (82.8 ± 12.6%) or to control subjects (96.7 ± 0.9%). With regard to TCR*ζ* MFI value, it is lower (Kruskall-Wallis test *P* = 0.001) in T cells from patients, irrespective of their anatomical location, whether tumoral (41.6 ± 21.4) or nontumoral (62.3 ± 16.6), when compared to control subjects (99.4 ± 21.4).

As before, CD3*ε* showed no significant differences.

Due to scarcity of tissue-derived T cells, the remaining experiments (Western blot, cDNA and mRNA analysis, and caspase-3 activity) were done only in T cells of blood origin. However, we may assume that the results obtained can confidently be extrapolated to tissue T lymphocytes based on the flow cytometry data.

#### 3.1.3. Western Blot

**Figure 2:**
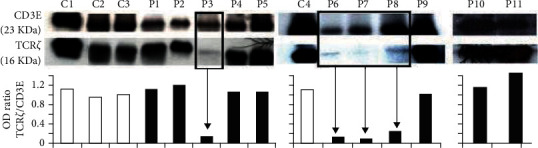
Western blot analysis of TCR*ζ* and CD3*ε* expression. Some patients (P3, P6, P7, and P8) showed reduced TCR*ζ* expression with an OD ratio below 0.3; others showed values comparable to those obtained in control subjects, C: control subjects, P: patients.

TCR*ζ* expression was also analyzed by Western blot.  Figure 2 shows the results obtained with 11 patients (not previously analyzed by flow cytometer) and 4 healthy subjects. In some patients (see P3, P6, P7, and P8), low TCR*ζ* levels were clearly observed, with a TCR*ζ*/CD3*ε* ratio below 0.3, whereas some other patients yielded results comparable to the ones obtained with control subjects. These results mirror flow cytometry data in that TCR*ζ* expression is defective in some, but not all, patients.

#### 3.1.4. cDNA Sequence

cDNA was obtained and sequenced in patients (*n* = 14) or healthy individuals (*n* = 8), to assess whether genomic differences could account for the defective TCR*ζ* expression (Table 2). Nine single-nucleotide polymorphisms (SNPs) were found. Six of them were already described (with no known relation to function, although, interestingly, the 1572 G to A transition is found in eleven of twelve patients and in three of five control subjects), whereas three new ones are reported in the present work: a C to T transition in position 373 and a G to A transition in positions 856 and 1542 (GenBank accession numbers EF364117, EF364118, and EF364119, respectively). The TAC to TAT transversion yields a synonymous change and will not influence TCR*ζ* levels. The remaining two are in the noncoding region of the gene (3′-UTR). Targetscan software analysis revealed that the latter (position 1542) could be target of miRNA MiR6832-5p and potentially modulate the posttranscriptional regulation of the gene. The elucidation of the functional relevance of these SNPs merits further consideration, increasing the number of patients studied.

#### 3.1.5. TCR*ζ* mRNA Levels

To assess whether TCR*ζ* mRNA levels mirrored the decreased protein expression found, qRT-PCR was carried out to measure specific TCR*ζ* mRNA levels. Mann-Whitney test disclosed no difference between the group of patients (*n* = 19; 0.58) and healthy individuals (*n* = 18; 0.99 p N.S., data not shown).

#### 3.1.6. Caspase-3 Activity

Since elevated caspase-3 activity has been involved in low TCR*ζ* expression in other pathologies (SLE), we measured this enzymatic activity in cell extracts. Patients (*n* = 6) showed higher activity (2.97 ± 1.9 pmol p-Na/min/*μ*g protein) than control subjects (*n* = 5, 1.02 ± 0.5 pmol p-Na/min/*μ*g protein, *P* = 0.009). Moreover, the use of the caspase-3 inhibitor Ac-DEVD-FMK in T cells of patients reduced by 70% the activity of the enzyme (*P* < 0.05 Wilcoxon test). However, TCR*ζ* expression was not recovered, suggesting that additional mechanisms must be involved in this expression defect.

## 4. Discussion

T lymphocytes are crucial to maintain cancerous progression in check [18], and defective cells will poorly carry out this task. The immune system tries to control tumor proliferation. T cells primed with tumoral antigens initiate a response aiming at eliminating tumoral cells (immunosurveillance). In this process, however, T cell activation is physiologically controlled (immune checkpoints), and this immunosuppression can promote tumor progression. In fact, cancer immunotherapy aiming at checkpoint inhibitors has gained support in recent years [19]. Low expression of the TCR*ζ* chain has been reported in several types of cancer, including head and neck cancer [20], ovarian carcinoma [21], renal cell carcinoma [22], prostate [23], colorectal carcinoma [24], melanoma [25], oral cancer [9], and, in the present work, gastric carcinoma.

We have measured the presence of this chain in enriched T cell populations and observed that the values obtained were consistently lower in patients. Thus, the percentage of TCR*ζ*-expressing cells was reduced in patients with cancer, as compared to healthy subjects, along with a significantly reduced MFI expression on the surface of T lymphocytes. This is true for blood- and tissue-derived T cells and is consistent with results obtained using different TCR*ζ*-specific monoclonal antibodies (results not shown). Regarding the TCR*ζ* expression in tissue-derived T cells from patients, two aspects merit further comment. First, the levels achieved were always lower than the ones obtained in their blood-derived counterparts, assessed either as percentage or as MFI values. This decrease was even more pronounced in T cells eluted from tumoral specimens. A similar finding was previously reported by our group [14]. Secondly, when compared to tissue-derived cells from healthy subjects instead, the protein expression level was consistently lower in patients, irrespective of the origin, tumoral or nontumoral, of the pieces used to isolate T cells. It is then clear that in patients, the expression of the TCR*ζ* chain is decreased, more in tissue than in blood and more in cancerous than in noncancerous regions of the tissue.

Western blot analysis (Figure 2) supports the cytometric results, as weaker bands are observed in the lanes corresponding to patients.

Our results match previous published data [26] that also reported diminished expression of this protein in the peripheral blood of some, but not all, gastric cancer patients, especially in patients with advanced disease. Given the size of our cohort, no sound statistical comparisons could be done if the group were split according to clinical status.

Although there are slight age differences between our groups of patients and healthy subjects, published work state that age differences between patients and control subjects does not affect CD3*ζ* expression levels. Thus, we can confidently accept the results obtained [27].

It remains unclear how this defective expression takes place in these cells, and two main types of mechanisms have been proposed: either a genetic defect underlies it or an exogenous substance (such as arginase or a tumor-derived factor) is the causative agent. However, this substance has not been clearly identified, though some reports have been published [21]. Since previous data from our group suggested that this defective expression could be inherent to the T cells of patients [15] it seemed adequate to study the TCR*ζ* cDNA sequence in patients and compare it to healthy subjects. Most of the sequences obtained matched already published sequences, although three new polymorphisms (see Table 2) were found, whose frequency, however, did not differ between the two groups studied. Thus, no genomic TCR*ζ* defect seems then responsible for this defective TCR*ζ* expression in patients.

We described a 9pb deletion in the 3′-UT region of the TCR*ζ* gene [28] in between two adenisone-uridine rich elements (AREs), in a patient with cutaneous angiosarcoma and gastric metastases. Interestingly, and according to http://www.targetscan.org/, this 9 bp sequence acts as potential target of two miRNAs (hsa-miRNA-767 and hsa-miRNA-4470), thus likely affecting protein expression [29]. Given the relevance of this region in the mRNA stability and, thus TCR*ζ* protein expression, this deletion may affect protein levels. In our cohort, only one of the patients and none of the control subjects analyzed presented this deletion.

Since no differences were found in the cDNA sequence, we then decided to test whether differences in the mRNA levels could explain our findings. However, qRT-PCR revealed no significant differences in the level of TCR*ζ* mRNA between patients and control subjects. Given that no changes in the mRNA molecule have been found (both the sequence and the levels are normal in the group of patients), and that our group [14] disclosed no differences in the promoter region of the gene, we felt that no further genomic (DNA) analysis (intron sequencing) was required.

We then went on analyzing caspase-3 activity, involved by some authors in low TCR*ζ* expression [6, 7]. In keeping with previous published data, our results revealed higher enzymatic activity in T cells from patients when compared to control subjects, activity which could be diminished (by 70%) using the Ac-DEVD-FMK inhibitor. However, and to some extent unexpectedly, CD3*ζ* expression does not recover in patients upon treatment with the inhibitor.

Altogether, these data seem to locate at the posttranscriptional level the defect responsible for the altered CD3*ζ* expression. Nevertheless, and according to the data herein presented, caspase-3 activity does not account for it, and further research is then required to unveil the mechanism linked to this CD3*ζ* low expression.

These results also resemble those published by Kulkarni et al. [9] in patients with oral cancer. They reported low TCR*ζ* expression in T cells, whether of blood origin or located at the tumor site. In the latter, this low expression was clearly due to transcriptional problems, whereas in the former, posttranslational problems accounted for the defect. Likewise, it has been reported in ovarian carcinoma that TCR*ζ* expression in T cells depended on the proximity to the tumoral mass [30]: when T cells were isolated from ascites fluid or peripheral blood, neither TCR*ζ* nor the corresponding mRNA transcript was affected, whereas in solid tumor infiltrating T lymphocytes both, the protein and the mRNA were absent.

Our group reported similar findings: stable gastric mucosa-derived T cell lines (HVS-transformed) displayed lower chain expression than peripheral blood derived T cell line [13]. Similarly, we show here that the loss of TCR*ζ* expression is more prominent in tissue-derived T cells (and more so in tumor sections) than in blood T cells, when compared to corresponding samples from control individuals. Unfortunately, caspase-3 activity was not tested in T lymphocytes of tissue origin, due to scarcity of samples.

Diminished expression of TCR*ζ* might affect the surface expression of the whole TCR-CD3 complex, since TCR*ζ* is the limiting factor in the assembly process [31], and it is then be conceivable to find a reduced CD3*ε* expression. In fact, published work [32] using immunoprecipitation of multiprotein complexes followed by flow cytometry (IP-FCM) revealed that a decreased TCR*ζ* expression affected the integrity of the TCR-CD3 complex. Previous reports revealed that TCR complexes lacking the TCR*ζ* chain are more rapidly endocytosed than full TCR complexes. TCR*ζ* seems then to stabilize the TCR complex on the cell surface [33].

However, and in keeping with other published data [34, 35], we detected just a minor reduction in the level of the CD3*ε* chain on the cell surface, a reduction that did not reach significance. Moreover, Western blot analysis (which detects total protein amount and not just membrane-bound) showed that in patients with low TCR*ζ* levels, CD3*ε* expression was like that of healthy individuals, indicating that its biosynthesis is not affected. Interestingly, previous analysis carried out on NK cells from SLE patients [6] revealed a diminished expression of CD3*ζ* with no effect on other molecules such as NKp30 and NKp46, which also signal via CD3*ζ*.

There are, however, some discrepancies in the literature with this regard. While some authors mention a concomitant diminished CD3*ε* and TCR*ζ* expression in rheumatoid arthritis [36], in response to TNF [37], in murine models of cancer [38], or in inflamed tissues [39], some others do not [5].

It must be stressed that in these latter instances, the absent TCR*ζ* is substituted in the TCR complex by the Fc*ε*R*γ* chain, a feature not found in our patients with gastric adenocarcinoma [14]; this could explain the lower activation state found in T cells from gastric cancer patients.

## Figures and Tables

**Table 1:** Primers used for TCR*ζ*-cDNA amplification.

Fragment (size)	Primer	Sequence	Sense
F1Z (296 pb)	F1FZ	CCTCTTTCTGAGGGAAAGGA	5′-3′
	F1RZ	CCACGTCTCTTGTCCAAAAC	5′-3′
F2Z (364 pb)	F2FZ	CGAGCTCAATCTAGGACGAA	5′-3′
	F2RZ	GTGAACCGGGTTGTAAATGC	5′-3′
F3Z (371 pb)	F3FZ	GGGGATTTCACCACTCAAAG	5′-3′
	F3RZ	CATTAGGGCATGTGCTAGCA	5′-3′
F4Z (381 pb)	F4FZ	CAGCTGAGTTGTTGAGTCTG	5′-3′
	F4RZ	CAGTCTGTTCATCTTCTGGC	5′-3′
F5Z (323 pb)	F5FZ	CGCACCATTGAACTGTACCA	5′-3′
	F5RZ	GAGCAGAGAGCGTTTTCCAT	5′-3′

**Table 2:** TCR*ζ* gene polymorphisms in 44 gastric cancer patients and 33 control subjects.

Genotype												
	WT/WT				WT/POL				POL/POL			
SNP	Controls		Patients		Controls		Patients		Controls		Patients	
	(*n*)	(%)	(*n*)	(%)	(*n*)	(%)	(*n*)	(%)	(*n*)	(%)	(*n*)	(%)
373 C/T^∗^	7	87.5	12	85.7	1	12.5	2	14.3	0	0.0	0	0.0
856 G/A^∗^	7	87.5	14	100.0	1	12.5	0	0.0	0	0.0	0	0.0
1235 C/G	8	100.0	14	100.0	0	0.0	0	0.0	0	0.0	0	0.0
1343 ins/G	6	75.0	11	78.6	2	25.0	2	14.3	0	0.0	1	7.1
1453 C/G	6	75.0	11	78.6	2	25.0	2	14.3	0	0.0	1	7.1
1460 T/A	6	75.0	9	64.3	2	25.0	3	21.4	0	0.0	2	14.3
1542 G/A^∗^	6	100.0	10	83.3	0	0.0	1	8.3	0	0.0	1	8.3
1553 A/T	6	100.0	12	100.0	0	0.0	0	0	0	0.0	0	0.0
1572 G/A	2	40.0	1	8.3	3	60.0	11	91.7	0	0.0	0	0.0

WT: wild type; POL: polymorphism; *n*: sample size SNP; ^∗^ new polymorphisms deposited at the GenBank with the accession numbers EF364119, EF364117, and EF364118, respectively.

## Data Availability

Data are available within the article on request from the authors.
